# Assessment of the Pharmacological Properties and Phytochemical Profile of *Bruguiera gymnorhiza* (L.) Lam Using In Vitro Studies, In Silico Docking, and Multivariate Analysis

**DOI:** 10.3390/biom10050731

**Published:** 2020-05-07

**Authors:** Nabeelah Bibi Sadeer, Kouadio Ibrahime Sinan, Zoltán Cziáky, József Jekő, Gokhan Zengin, Rajesh Jeewon, Hassan H. Abdallah, Kannan R. R. Rengasamy, Mohamad Fawzi Mahomoodally

**Affiliations:** 1 Department of Health Sciences, Faculty of Science, University of Mauritius, 230 Réduit, Mauritius; nabeelah.sadeer1@umail.uom.ac.mu (N.B.S.); r.jeewon@uom.ac.mu (R.J.); 2 Department of Biology, Science Faculty, Selcuk University, 42130 Konya, Turkey; sinankouadio@gmail.com (K.I.S.); gokhanzengin@selcuk.edu.tr (G.Z.); 3 Agricultural and Molecular Research and Service Institute, University of Nyíregyháza, 4400 Nyíregyháza, Hungary; cziaky.zoltan@nye.hu (Z.C.); jjozsi@gmail.com (J.J.); 4 Chemistry Department, College of education, Salahaddin University-Erbil, 44001 Erbil, Iraq; hwchems@gmail.com; 5 Department of Bioresources and Food Science, College of Life Sciences, Konkuk University, Seoul 05029, Korea; rengasamy@iceir.net; 6 Department for Management of Science and Technology Development, Ton Duc Thang University, Ho Chi Minh 758307, Vietnam; 7 Faculty of Applied Sciences, Ton Duc Thang University, Ho Chi Minh 758307, Vietnam

**Keywords:** mangrove, Mauritius, multivariate analysis, phytochemicals, enzymatic effects, docking, antioxidants

## Abstract

*Bruguiera gymnorhiza* (L.) Lam. is claimed to effectively manage a number of ailments including diabetes and associated complications. Nonetheless, no attempt has been made to delineate its pharmacological propensities and phytochemical profile. This study was designed to appraise the antioxidant and enzymatic inhibitory properties relevant to the management of diabetes mellitus, obesity, and neurodegenerative and skin disorders. A combination of colorimetric assays and ultra-high-performance liquid chromatography/electrospray ionization tandem mass spectrometry (UHPLC-ESI-MS/MS) were applied for the phytochemical screening of leaf, root, twig, and fruit extracts (methanol and ethyl acetate). In vitro antioxidant evaluations were via radical scavenging abilities (DPPH, ABTS), reducing potential (FRAP, CUPRAC), chelating power, and total antioxidant capacity (phosphomolybdenum). Seven key metabolic enzymes (α-amylase, α-glucosidase, tyrosinase, elastase, lipase, AChE, and BChE) were targeted to determine the inhibitory effects. Multivariate and in silico docking analysis were performed on collected data. Methanolic fruit extract yielded the highest total phenolic, tannin, and triterpenoid contents (174.18 ± 4.27 mg GAE/g, 176.24 ± 3.10 mg CE/g, 63.11 ± 3.27 mg OAE/g, respectively); significantly depressed tyrosinase, elastase, and α-amylase activities (155.35 ± 0.29 mg KAE/g, 4.56 ± 0.10 mg CAE/g, 1.00 ± 0.05 mmol ACAE/g, accordingly); and harboured the most potent antioxidant capacities with DPPH, CUPRAC, FRAP (492.62 ± 5.31, 961.46 ± 11.18, 552.49 ± 8.71 mg TE/g, respectively), and phosphomolybdenum (4.17 ± 0.31 mmol TE/g) assays. Multivariate analysis suggested that the type of solvents used influenced the biological activities more compared to plant parts. Docking analysis showed that azelaic acid binds with tyrosinase by Van der Waals and conventional hydrogen bonds. We anticipate that the present study may establish baseline data on this halophyte that could open new avenues for the development of biomedicine.

## 1. Introduction

There are up to 8.7 million species from both plant and animal kingdoms. The kingdom Plantae itself englobes an estimated number of 391,000 vascular plant species, and Allkin Bob from the Royal Botanic Gardens, Kew, located in London, United Kingdom, has made a global classification recording 28,187 plants as medicinally important [1,2,3]. Recently, Frances Hamilton Arnold, a Nobel prize winner in chemistry in the year 2018, stated “Nature is the best chemist of all time” [4]. Indeed, this array of plant species from nature has always proved their worth in the medical lore since the dawn of human civilization. For instance, *Cupressus sempervirens* L. is the oldest medicinal plant, known since 2600 BC, and is still being used against colds and inflammation [5]. It is worth mentioning that around 11% of the 252 drugs approved by the Food and Drug Administration (FDA) originate from flowering plants [6], which undoubtedly makes nature indeed the best chemist of all time. 

The present paper aims at scrutinizing a poorly explored mangrove plant—*Bruguiera gymnorhiza* (L.) Lam. (*B. gymnorhiza*)—usually found along the coastlines of Mauritius. Morphologically, *B. gymnorhiza* is a shrub reaching a height of 5–8 m with pneumatophores as a root system. The plant has rough, reddish-brown bark; large, dark green, and elliptical leaves with reddish petioles blooming creamy-white to brown flowers; and produced green cigar-shaped propagules [7]. This mangrove plant has a deep-rooted use in traditional medicine, where it is used to manage diabetes and hypertension in Mauritius and commonly used in Asian countries against several other diseases, including diarrhoea, viral fever, ulcers, shingles, and haematuria [7,8,9,10]. 

Despite folk belief from different ethnic groups, the medicinal properties of the halophyte are poorly understood in the modern scientific community due to a lack of thorough pharmacologically cogent evidence. Consequently, to bridge the research gap between ethnomedicinal uses of *B. gymnorhiza* and modern pharmacotherapy is the endeavour of this study. From past literature, *B. gymnorhiza* has displayed promising pharmacological activities. For instance, Barik et al. screened the methanolic leaf extract for possible anti-inflammatory activities. The obtained results in the study showed that, at a dosage of 100 µg/mL, 65.1% inhibition was reported [11]. To date, few studies have been conducted to evaluate the antioxidant properties of the mangrove plant. For instance, an ascorbic acid equivalent of 1.55 mg/g and 1.25 mg/g were noted for the methanolic leaf and root extracts, respectively, with a ferric acid reducing power (FRAP) assay [12]. Furthermore, the plant was screened for its antimicrobial properties by Haq et al. Accordingly, the methanolic leaf extract inhibited the growth of *Bacillus cereus, Staphylococcus aureus, Escherichia coli*, and *Pseudomonas aeruginosa*, resulting in inhibition zones of 12.67, 14.34, 8.87 and 7.85 mm, respectively [13]. In vitro anti-cancer study was conducted on four types of mangrove species—namely *B. gymnorhiza, Aegiceras corniculatum* (L.) Blanco, *Aegialitis rotundifolia* Roxb., and *Lumnitzera racemosa* Willd.—against the melanoma cell line HepG2. Findings revealed that the methanolic extract of *B. gymnorhiza* exhibited the highest cytotoxicity activity with the lowest IC50 value (–77.29) at a concentration of 200 µg/mL. The study concluded that *B. gymnorhiza* can be a source for new lead structures for the design of anti-cancer drugs [14]. Another study showed that the aqueous extract demonstrated cytotoxicity effects against breast ductal carcinoma cells (MDA-MB-435S) with an IC50 value of 1.38 mg/mL [15]. The method used to conduct such cytotoxicity testing on these kind of plant extracts is the *Allium cepa* test [16].

These fragmented existing studies attempting to cover the pharmacological aspects of the plant are insufficient, unsystematic, and inconclusive. This scarcity of knowledge on the medicinal properties and phytochemical composition of *B. gymnorhiza* was thus an added impulse in the compilation of the present paper. The work is presented in three-fold—to (1) characterize the phytoconstituents using ultra-high-performance liquid chromatography/electrospray ionization tandem mass spectrometry (UHPLC-ESI-MS/MS) and quantify common bioactive compounds using in vitro assays, (2) validate its biological activities in terms of antioxidant activities (six assays) and enzyme inhibitory effects (seven enzymes), and (3) analyse the collected data using multivariate and in silico docking analysis. To the best of our knowledge, this work has no parallel in the whole corpus of existing scientific data on *B. gymnorhiza*.

## 2. Materials and Methods

### 2.1. Collection of Plant Materials and Extraction

The plant materials of *B. gymnorhiza*—namely leaves, twigs, roots, and fruits—were collected at Bambous Virieux in Mauritius (GPS: 20°20′13.88″ S; 57°45′54.99″ E) in April 2018. The collected materials were identified by a botanic specialist of the Mauritius Herbarium at the Mauritius Sugarcane Industry and Research Institute (MSIRI) located in Réduit, Mauritius. A voucher specimen bearing the reference number MAU 0029125 was deposited at the herbarium. The plant parts were allowed to dry in a well-ventilated area away from direct sunlight after being thoroughly washed under running tap water to remove all surface debris. After a constant mass was recorded, the dried raw materials were pulverized, and each powdered plant samples, namely leaves, roots, twigs, and fruits (50 g), were separately soaked in 500 mL of methanol (70%) and ethyl acetate, respectively for nine days with constant shaking after every 24 h. The mixtures were filtered using Whatmann filter paper grade 1 and concentrated in a rotary evaporator at low temperature and pressure. The crude extracts were then stored at 4 °C in the dark until further analysis.

### 2.2. Profile of Bioactive Compounds

The total bioactive compounds—namely total phenolic (TPC), flavonoid (TFC), phenolic acid (TPA), flavanol (TFlavaC), condensed tannins (TTC), and triterpenoids (TTriC)—were determined colorimetrically as previously described [17,18,19]. The results were expressed as mg of standard compounds (gallic acid for TPC; rutin for TFC; caffeic acid for TPA; catechin for TFlavaC and TTC; oleanolic acid for TTriC) per g of dried extract.

Chemical compositions of the *B. gymnorhiza* extracts were determined using a Dionex Ultimate 3000RS UHPLC instrument (Thermo Scientific, MA, USA). The extracts were filtered through 0.22 μm PTFE syringe filter (Labex Ltd, Hungary) before High Performance Liquid Chromatography (HPLC) analysis. The compounds were separated on a Thermo Accucore C18 (Thermo Scientific, MA, USA) (100 mm × 2.1, mm i. d., 2.6 μm) column thermostated at 25 °C (±1 °C). The solvents used were water (A) and methanol (B), both acidified with 0.1 % formic acid. The flow rate was maintained at 0.2 mL min^−1^. The elution gradient was isocratic 5 % B (0–3 min), a linear gradient increasing from 5% B to 100% (3–43 min), 100% B (43–61 min), a linear gradient decreasing from 100% B to 5% (61–62 min), and 5% B (62–70 min). The column was coupled to a Thermo Q Exactive Orbitrap mass spectrometer (Thermo Scientific, MA, USA) equipped with electrospray ionization source. Mass spectrometry (MS) spectra were recorded in positive and negative-ion mode, respectively.

Trace Finder 3.1 (Thermo Scientific, MA, USA) software was applied for target screening. The compounds listed in Table 1, Table 2, Table 3 and Table 4 were identified on the basis of our previous published works or data found in the literature using exact molecular mass, isotopic pattern, and characteristic fragment ions. In every case, the exact molecular mass, isotopic pattern, characteristic fragment ions, and retention time were used for the identification of the compounds.

### 2.3. Determination of Antioxidant and Enzyme Inhibitory Effects

The metal chelating (MC), phosphomolybdenum (PPBD), ferric reducing power (FRAP), cupric reducing antioxidant capacity (CUPRAC), 2,2′-azino-bis (3-ethylbenzothiazoline-6-sulphonic acid) (ABTS), and 2,2-diphenyl-1-picrylhydrazyl (DPPH) activities of the extracts were evaluated following the methods described by Grochowski et al. (2017) [20]. The antioxidant activities were reported as Trolox equivalents, whereas ethylenediaminetetraacetic acid (EDTA) was used for metal chelating assay. The possible enzymatic inhibitory activities of the extracts against acetylcholinesterase (AChE), butyrylcholinesterase (BChE) (by Ellman’s method), tyrosinase, α-amylase, α-glucosidase [21], elastase [22], and lipase [20] were assessed using standard in vitro bioassays. 

### 2.4. Statistical Analysis

One-way analysis of variance (ANOVA) with post-hoc Tukey test was conducted to determine significant differences between the extracts (*p* < 0.05). Pearson correlation and chord diagram were performed to assess the interaction between bio-compounds and biological activities (antioxidant and enzymatic inhibition). Sparse Partial Least Squares (sPLS-DA) analysis was also done to evaluate the effect of the different extraction solvents (methanol and ethyl acetate) on the observed biological activities. The statistical procedures were performed using R software v. 3.5.1 (R Core Team, Vienna, Austria). The chord diagram was generated using Chordial software (San Francisco, USA).

### 2.5. In Silico Docking Calculations

Three bioactive compounds—namely, luteolin, taxifolin, bruguierol A, and brugierol—were selected for docking against lipase enzyme, while azelaic acid and quinic acid were docked against tyrosinase enzyme. The chemical structures of these inhibitors were downloaded from available online databases (PubChem and ChemSpider). The structures were then optimized to the ground state using the AM1 semiempirical method using VegaZZ software (Alessandro Pedretti, Milano, Italy). The crystal structures of lipase and tyrosinase enzymes were downloaded from Protein Databank RCSB (pdb code 5I38 for tyrosinase and 1LPB for lipase enzyme). AutodockTools-1.5.6 (The Scripps Research Institute, La Jolla, CA, USA) was used to prepare the docking input files while Autodock4 software was used to perform the docking calculations using Lamarckian genetic algorithm. Two hundred and fifty conformations were docked, clustered, and ranked according to the binding free energy. The enzyme-substrate interactions were elucidated and visualized using Discovery Studio 5.0 visualizer (Dassault Systems BIOVIA, CA, USA).

## 3. Results

### 3.1. Bioactive Compounds

Plants have been providing humanity with multiple molecules of medicinal importance for ages, exhibiting numerous biological activities, including antioxidant, anticancer, anti-inflammatory, neuroprotective, and antimicrobial activities, among others [23]. Therefore, the quantitative preliminary screening of phytochemicals of the different plant extracts of *B. gymnorhiza* was undertaken. The studied extracts were evaluated for their phenolic, phenolic acid, flavonoid, flavanol, condensed tannin, and triterpenoid content using colorimetric methods. The results are summarized in Table 5.

The methanolic extracts yielded the highest quantity of bioactive compounds, in contrast to ethyl acetate extracts, except for flavonoid content, whereby the ethyl acetate fruit extract possessed the highest amount (36.64 ± 0.50 mg RE/g) expressed as equivalents of rutin. It is reported that less polar flavonoids are extracted by less polar solvents—namely dichloromethane, chloroform, and ethyl acetate, among others [24]. Thus, it could be hypothesized that the type of flavonoid present in our extracts might be less polar. 

The methanolic fruit possessed the highest total phenolic content of 174.18 ± 4.27 mg GAE/g, while the ethyl acetate twig showed the lowest amount at 27.27 ± 0.43 mg GAE/g (Table 5). It is interesting to highlight that the phenolic content was quantified in different amount from the same type of extract by different studies. For instance, a study conducted by Haq et al. quantified 178.73 ± 0.23 mg GAE/g of phenolic content from the methanolic leaf sample [13], while Karim et al. (2020) reported a phenolic content equivalent of 58.917 ± 0.601 mg gallic acid/g [25] and Banerjee et al. (2008, reported a much lower quantity of 8.25 ± 0.31 mg GAE/g from the same extract (i.e., methanolic leaf) [12]. Herein, our results showed that the methanolic leaf sample yielded a lower amount (30.26±0.25 mg GAE/g) compared to the studies of Haq et al., (2011) and Karim et al., (2020) but higher than Banerjee et al., (2008). This situation is favourable to explain that the production of polyphenols in plants is influenced by a number of environmental factors namely light intensity, carbon dioxide concentration, maturity of plant parts and plant age [26]. The methanolic root was found to be most abundant in terms of phenolic acid, flavanol and triterpenoid contents with equivalent values of 9.86 ± 0.37 mg CAE/g, 119.00 ± 1.49 mg CE/g and 55.73 ± 6.48 mg OEA/g, respectively. Additionally, among the prepared extracts, methanolic fruit was richer in tannin (176.24 ± 3.10 mg CE/g) and triterpenoid contents (63.11 ± 3.27 mg OAE/g) (Table 5).

**Figure 1:**
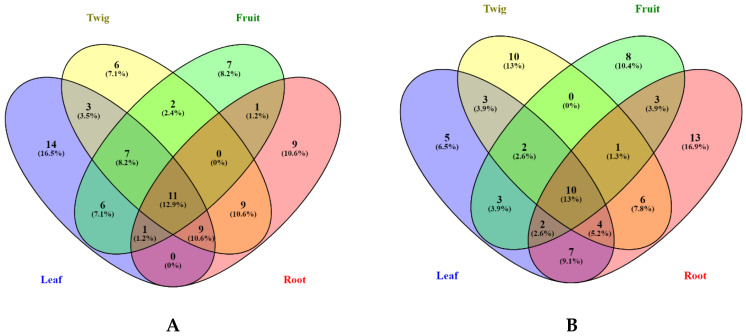
Venn diagrams showing number of compounds identified from methanolic (**A**) and ethyl acetate (**B**) extracts.

However, it is argued that the total bioactive compound assays provide only a basic insight on the phytochemical content present in plant extracts in contrast to a full phytochemical profile provided by extensive techniques. Indeed, in this study we screened the different extracts using UHPLC-ESI-MS/MS. Table 1, Table 2, Table 3 and Table 4 detailed the types of phytochemicals identified from the different extracts of *B. gymnorhiza*. Our results demonstrated that both methanolic and ethyl acetate extracts of *B. gymnorhiza* possessed the same number of compounds (36) (see Figure 1) despite numerous publications acknowledged that methanol is the most efficient extraction solvent [22]. Nonetheless, the chemical composition of the extracts showed wide variability within the different plant parts irrespective of the extraction solvents used. For instance, the Venn diagrams illustrated in Figure 1 showed that methanolic leaf extract yielded 14 different compounds, in contrast to only five compounds identified in the ethyl acetate leaf extract. Six compounds were identified in the methanolic twig extract, while 10 were identified in the ethyl acetate twig extract. The same holds true for the other two plant parts—the fruit and root, as shown in Figure 1. Eleven compounds were found in common in the methanolic extracts of the four plant parts, while 10 were common in the four ethyl acetate extracts.

### 3.2. Antioxidant Assays

Reactive derivatives of oxygen, also known as reactive oxygen species (ROS), are usually produced from local metabolic processes catalyzed by redox enzymes [27]. As a normal defensive mechanism, our body neutralize the presence of ROS with the help of antioxidants to maintain a healthy balance. However, in some cases, the balance is disrupted due to an overproduction of ROS and an ineffective antioxidant defense system, leading to the initiation of chronic diseases—namely, cancer, neurodegenerative and cardiovascular diseases, inflammatory disorders, and diabetes [27,28,29]. As such, a need for antioxidants through dietary supplements is highly required. In this context, we screened our extracts for potential antioxidant properties using three different mechanisms—radical quenching, reducing potential, and metal chelating.

Our present research aims at quantifying the antioxidant capacity of the methanolic and ethyl acetate extracts of various parts (leaves, twigs, roots, fruits) of *B. gymnorhiza* using six assays. The results given in Table 6 showed that the methanolic extracts exhibited higher level of antioxidant power compared to the ethyl acetate extracts. 

**Figure 2:**
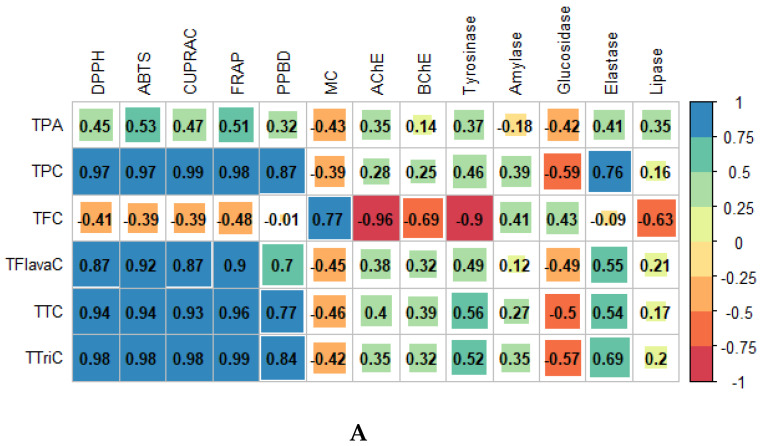

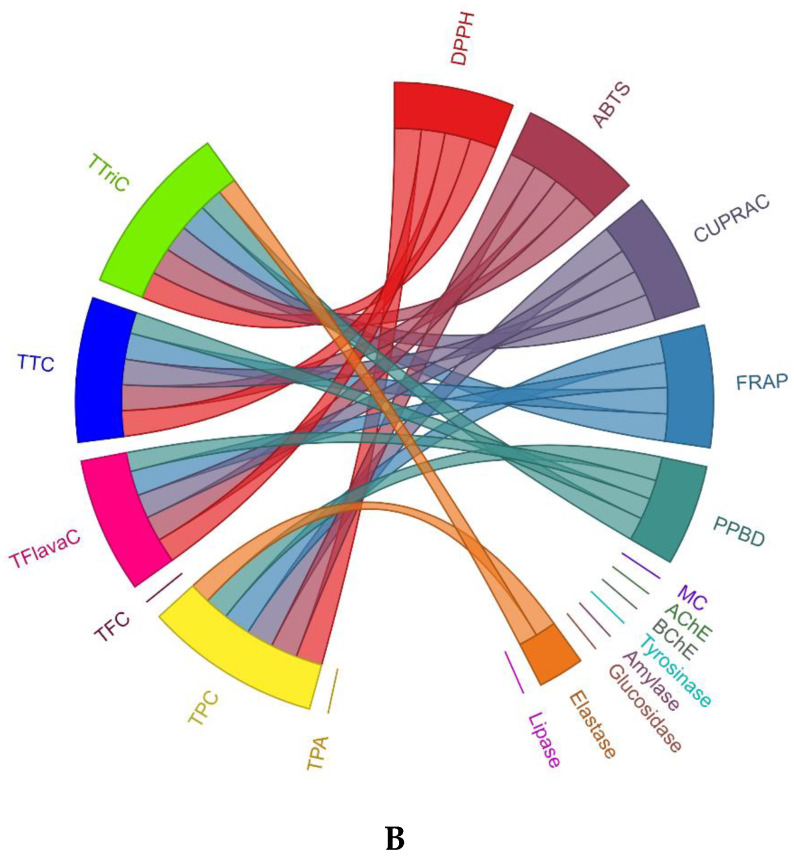
(**A**) Pearson correlation and (**B**) Chord diagram showing correlation between bio-compounds and biological activities. The connecting chord represents a Pearson’s correlation coefficient greater than 0.6.

A study conducted by Dailey et al. revealed that extraction solvents have a significant impact on the antioxidant capacities of plant extracts since the extraction of bioactive compounds depends on the type of solvent used [30]. Indeed, data analysis through Pearson correlation coefficient (R) and the illustrative chord diagram clearly showed that most bio-compounds were positively correlated with most antioxidant assays in contrast to enzymatic assays, which revealed lower R values (see Figure 2A,B). For instance, all quantified bio-compounds displayed a coefficient value < 0.6 with six enzymes namely AChE, BChE, tyrosinase, amylase, glucosidase, and lipase showing a weak association between these two entities (i.e., bio-compounds and enzymes). However, only elastase enzyme showed an association of R > 0.6 with TPC and TTriC, as shown in the chord diagram (Figure 2B). Thus, it can be said that the bio-compounds such as phenolic acid, flavonoid, phenolic, tannin, flavanol, and triterpenoid have a stronger influence on the antioxidant assays than on the enzymatic assays. For instance, the Pearson correlation coefficient between triterpenoids and DPPH, ABTS, CUPRAC, and FRAP were 0.98, 0.98, 0.98, and 0.99, respectively, while lower R values were reported with AChE (R = 0.35), BChE (R = 0.32), tyrosinase (R = 0.52), amylase (R = 0.35), glucosidase (R = –0.57), elastase (R = 0.69) and lipase (R = 0.20). Among the bio-compounds screened, triterpenoids have the strongest influence on the antioxidant assays since they showed the strongest Pearson correlation coefficient (R = 0.98) with most assays. However, a negative relationship was noted between most bio-compounds and metal chelating, except in the case of flavonoid, which demonstrated a relatively high R value of 0.77 (see Figure 2A). 

As shown in Table 6, methanolic fruit demonstrated the highest antioxidant potential. For instance, the extract proved to be the best DPPH scavenger (492.62 ± 5.31 mg TE/g), having the most powerful reducing abilities (552.49 ± 8.71 and 961.46 ± 11.18 mg TE/g with FRAP and CUPRAC assays, respectively) and possessed the highest total antioxidant capacity (4.17 ± 0.31 mmol TE/g). It is important to highlight that the methanolic fruit extract yielded the highest total phenolic content, a class of phytochemical known to have potent antioxidant effects [31]. However, the latter extract was not the most potent ABTS scavenger. This might be attributed to the fact that since DPPH and ABTS are two different radicals, their mechanism of reaction with phytochemicals are different [28]. Furthermore, the high antioxidant activities could be attributed to synergism operating between different bio-compounds. Indeed, the literature supports the idea that a combination of rutin and chlorogenic acid (which are identified in the methanolic fruit extract) display efficient synergistic effects [32]. The metal chelating ability of the extracts was also testified to, and a different trend was observed, whereby the ethyl acetate twig extract exhibited better chelating properties compared to the four different methanolic extracts. Our findings show that the ethyl acetate twig extract exhibited a significant ability to chelate ferrous ions with an EDTA equivalent value of 60.22 mg, while the highest value recorded from methanolic extracts was 30.84 mg EDTAE/g. The ferrous ion (Fe^2+^) has the capacity of generating free radicals by gaining or losing electrons. An abnormal level of metal ions may lead to numerous anomalies in our biological system. For instance, Fe^2+^ ions greatly contribute to oxidative damage, resulting in neurodegenerative complications [33]. Hence, searching for natural phytochemicals with the ability to chelate transition metal effectively could be therapeutically important [34].

### 3.3. Enzymatic Inhibitory Effects

Over 95% of the world’s population are suffering from health complications, and 2–3 billion people are encountering more than five illnesses, as stated by the Global Burden of Disease study [35]. This startling number of sick individuals around the world has triggered an urgent need in the pharmaceutical industry to develop novel and efficient medications for a healthier future. In fact, the etiology of diseases arises from the enzymes present in the human body [36]. For example, AChE is an enzyme responsible for lowering the level of acetylcholine in the brain, which eventually causes neurological disorders [37]. Thus, the inhibition of key enzymes responsible for various aliments are acknowledged to be a good strategy in the treatment of pathologies (neurodegenerative disorders: cholinesterase; diabetes mellitus: α-amylase and α-glucosidase; skin disorders: tyrosinase and elastase; obesity: lipase inhibition) [38,39]. Therefore, the quest to develop novel enzyme inhibitory agents is one of the most challenging ongoing research topics. Medicinal plants are the raw materials placed under the spotlight by most pharmaceutical industries, since it is claimed that these plants produced phytochemicals with the capacity to inhibit enzyme activities [36]. To join the current upsurge interest and to contribute in the quest of novel enzyme inhibitory agents, the investigations conducted herein are thus predominantly focused on the inhibition of cholinesterases (AChE and BChE), α-amylase, α-glucosidase, lipase, tyrosinase, and elastase by the extracts of *B. gymnorhiza*. Our results are summarized in Table 7. To the best of our knowledge, this is the first report assessing the inhibitory potentials of the leaves, roots, twigs, and fruits of *B. gymnorhiza* related to diabetes mellitus, neurodegenerative complications, skin disorders, and obesity.

A variation in the enzymatic activities was observed among the tested extracts. For instance, extracts showing potent activities with one enzyme displayed weak inhibition with other enzymes. This may be due to the presence of different phytochemicals exhibiting different enzymatic inhibitory properties. For instance, quercetin (flavonol) exhibits high degree of inhibition towards collagenase enzyme while apigenin (flavone) demonstrates a weak inhibition [40]. Herein, the ethyl acetate fruit extract yielding the highest total flavonoid content (Table 1), showed high α-amylase and α-glucosidase inhibitory effects (0.93 ± 0.04 and 28.59 ± 0.08 mmol ACAE/g, respectively) but weak tyrosinase (33.48 ± 0.43 mg KAE/g) and BChE (0.87 ± 0.03 mg GALAE/g) activity. Indeed, the interconnection between flavonoids and antidiabetic activities has been proven by a number of publications reporting flavonoids as promising polyphenolic compounds to successfully regulate food digestion by inhibiting both α-amylase and α-glucosidase enzymes [41,42,43], and similar findings are reported herein. 

Diabetes mellitus (DM) was first confirmed by Dobson in the 1770s. Regardless of the time elapsed, the mechanism of action of DM has not been fully unravelled [44]. The International Diabetes Federation (IDF) guidelines suggest that, in order to minimize the risk of developing further health complications caused by DM, glycated haemoglobin (HbA1c) should be well-below 7% (53 mmol/mol) [45]. However, a study conducted in China revealed that only one-third of diabetic patients under oral antidiabetic medications achieved the recommended HbA1c level (<7%), leading to a situation called “resistant diabetes”. Insulin injection is the next option to treat patients with “resistant diabetes”. However, this option represents discouraging after-effects regarding hypoglycaemia and weight gain [46]. Consequently, to develop novel medications to reverse poorly controlled type 2 DM is of extreme necessity. In the present research study, the methanolic fruit extract was the most potent α-amylase inhibitor but simultaneously no inhibitory activity was noticed against α-glucosidase. In fact, no activity was noted with all methanolic extracts. As stated in a review compiled by Doukyu and Ogino, certain organic solvents may cause inactivation and instability of enzymes, which could explain our results obtained from methanolic extracts [47] (Table 7). Nevertheless, the ethyl acetate extracts significantly depressed α-glucosidase activity with root and twig reported as the two most potent inhibitors (30.43 ± 0.07 and 30.98 ± 0.01 mmol ACAE/g, respectively). The *B. gymnorhiza* extracts were also screened for their anti-lipase activity since obesity and diabetes are believed to have an interlinked mechanism. The ethyl acetate leaf extract was reported as the most potent lipase inhibitor, with 102.80 ± 7.25 mg OE/g.

Tyrosinase is the prime enzyme involved the o-hydroxylation of monophenols and oxidation of o-diphenols to o-quinones resulting in the biosynthesis of melanin (brown or black pigment) [48]. Melanin has multifaceted roles; apart from giving colour to the skin, it also acts as a skin photo-protector and convert harmful UV light into harmless heat. Despite its good side, an overproduction of melanin leads to numerous undesirable skin problems including melanoma, lentigo, and post-inflammatory hyperpigmentation frequently caused by eczema, acne, and psoriasis [49,50]. Melanin also has a questioning side. Studies have claimed that melanin is highly correlated with advanced skin-related problems and is believed to protect cancerous cells from chemo-, radio-, and photodynamic therapy [51]. Additionally, the regular cellular metabolism may be subjected to metamorphism, and the probability of generating a mutagenic environment is elevated due to melanogenesis [52]. Thus, inhibition of tyrosinase activity is of major concern since several skin disorders may be controlled. Concerning our anti-tyrosinase activity of the extracts, the methanolic fruit extract was found to possess marked tyrosinase and elastase activities, with its anti-tyrosinase activity (155.35 ± 0.29 mg KAE/g) being notably stronger than its anti-elastase activity (4.56±0.10 mg CAE/g). These skin-aging-associated enzyme inhibitory activities are associated with the elevated antioxidant capacity of the extract, as previously discussed. Furthermore, this result might be attributed to the presence of the compounds catechin and chlorogenic acid in our extract, since they are known to suppress aging activity (Table 2) [53]. The intricated synergism operating between the compounds could also be responsible for the observed inhibition. 

In this work, the methanolic and ethyl acetate extracts were also assessed for their antiacetyl- and butyryl-cholinesterase activities. Both AChE and BChE enzymes are responsible for lowering the level of acetylcholine in the synaptic cleft of the brain leading to neurodegenerative disorders—namely, Alzheimer’s disease, Parkinson disease, and cognitive problems. Thus, searching for inhibitors of these enzymes should remain perennial since no cure has yet been registered for the aforementioned pathologies [37,54]. Our findings show that the ethyl acetate twig extract significantly inhibited both AChE and BChE enzymes with 5.34 ± 0.17 mg GALAE/g and 6.90 ± 0.10 mg GALAE/g, respectively. It is important to highlight that the methanolic extracts displayed relatively similar anti-AChE activities, while the ethyl acetate extracts depressed AChE enzymes differently. This result is in accordance with existing literature supporting the potent ability of coumarin (a compound presents only in the ethyl acetate twig extract) (Table 5) to inhibit AChE and BChE enzymes [54]. 

### 3.4. Multivariate Analysis

**Figure 3:**
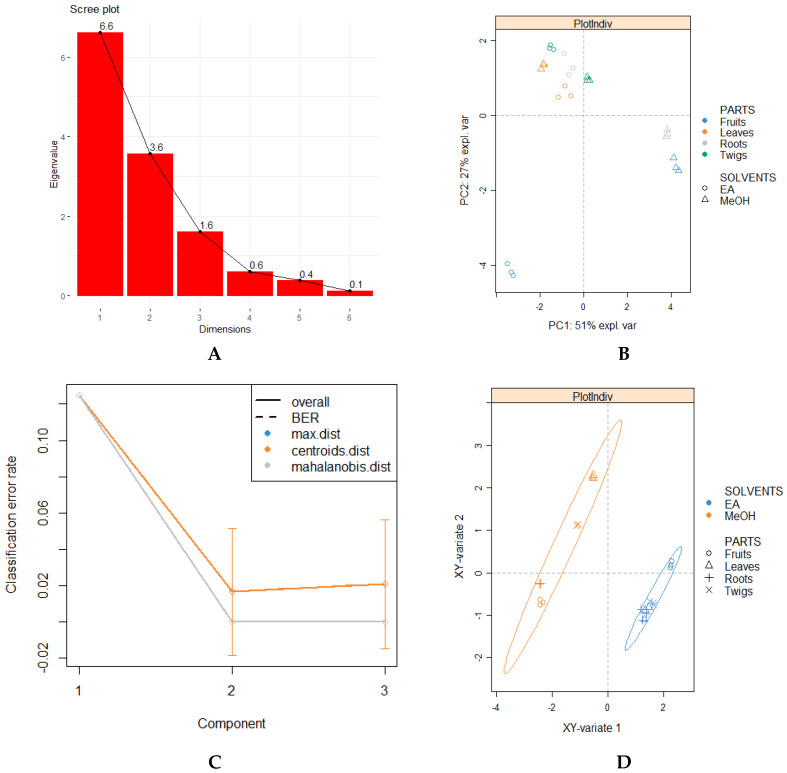

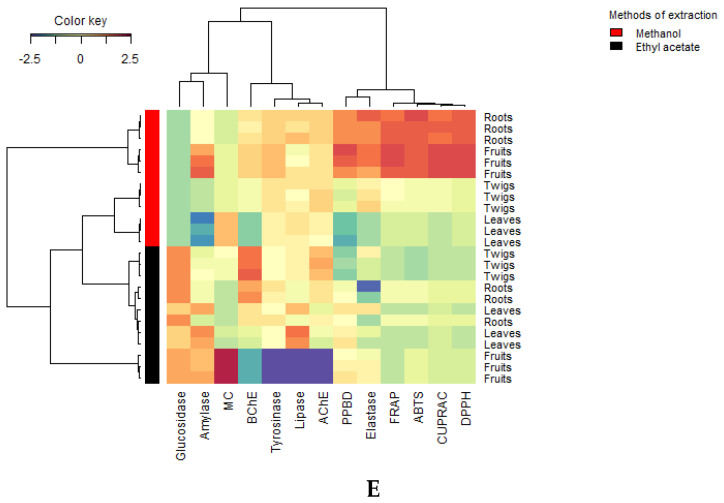
(**A**) Scree plot; (**B**) factorial plan 1–2 of the principal component analysis (PCA); (**C**,**D**) performance plot and Sparse Partial Least Squares (sPLS-DA) plot from results obtained from biological activities; (**E**) hierarchical cluster analysis shown as a heatmap.

A multivariate analytical approach was used to assess the influence of the two independent variables (plant parts and solvents) simultaneously on each dependent variable (bio-compound profiling and biological activities). As a first step, a scree plot was constructed to determine the number of significant components or factors to retain based on the Guttman–Kaiser criterion, which suggests that we consider only components with eigenvalues superior to one as important [55]. Herein, the first three principal factors are retained, since their eigenvalues are > 1, as illustrated in the scree plot (Figure 3A). In order to obtain an overview of the influence of plant parts and solvents on biological activities, principal component analysis (PCA) was performed. However, the application of PCA (unsupervised analysis) did not reveal significant differences between the samples since no clear-cut separation was observed (Figure 3B). Thus, sparse partial least squares discriminant analysis (sPLS-DA) (supervised analysis) was conducted since it enables the selection of the most discriminative features in a set of data projecting a clearer classification of the variabilities in contrast to PCA [56]. Prior to the sPLS-DA analysis, we graphed a performance plot by determining BER (balanced error rate) using both “centroids and mahalanobis” as prediction distance and five-fold cross-validation repeated 10 times to confirm the number of components to retain. As illustrated in Figure 3C, a steep decline was observed from component 1 to component 2, suggesting that “ncomp=2” was sufficient to achieve the best classification performance. 

Indeed, by retaining two components, the sPLS-DA plot provides a sharper separation of the extracts of *B. gymnorhiza*, resulting in two distinctly separated clusters based on the solvents used. In both clusters, all extracts prepared from different plant parts were clustered together, thus highlighting their low discriminating feature (Figure 3D). On this note, we could argue that the biological activities (antioxidant capacities and enzymatic effects) were greatly influenced by the type of solvents rather than the different plant parts used. Interestingly, similar classification was observed with the heatmap pattern (Figure 3E).

### 3.5. In Silico Docking

In order to study the inhibition affinity of the selected compounds, docking simulation was used to find the best conformation of these inhibitors and explain their interactions with the active site of the target enzyme. The list of compounds with their 2D chemical structures is shown in Table 8. The top ranked conformation with the highest binding affinity for each inhibitor is listed in Table 9. As shown in Table 9, luteolin has the highest binding affinity against lipase enzyme (–8.49 kcal/mol), and azelaic acid has the highest affinity against tyrosinase enzyme (–5.35 kcal/mol).

**Figure 4:**
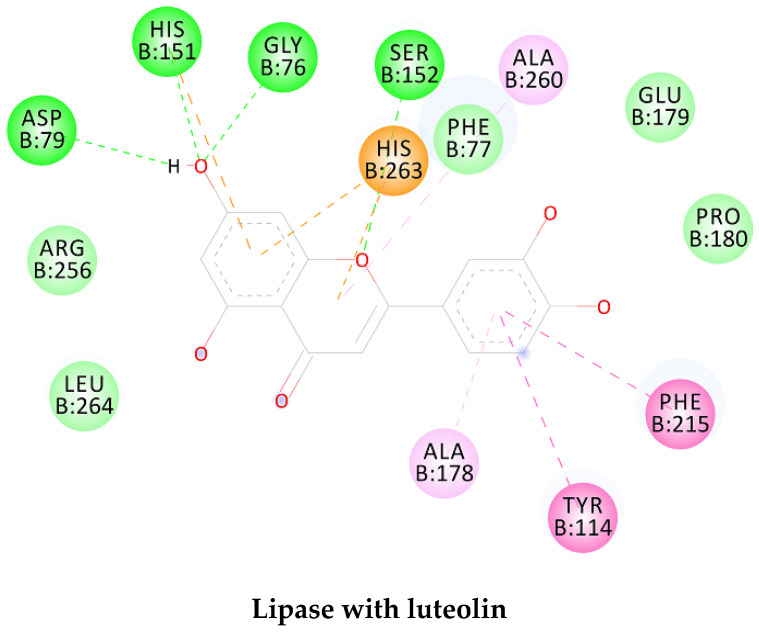

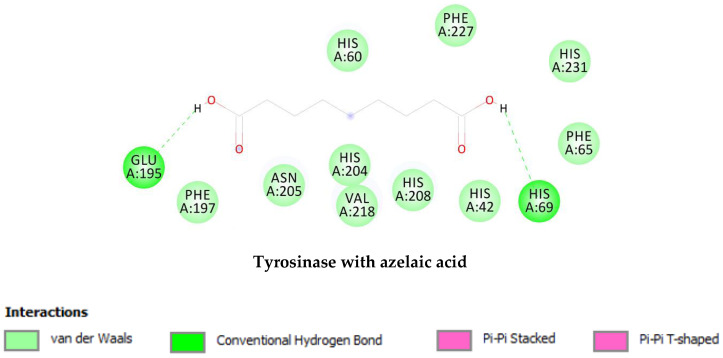
Enzyme-substrate interactions of luteolin and azelaic acid with lipase and tyrosinase enzymes, respectively.

The structure activity relationship for the docked compounds is elucidated in Figure 4. Luteolin has shown a variety of interactions with enzyme-active sites. Hydrogen bonds and pi–pi interactions are the strongest and in charge of the high binding affinity of the inhibitor. On the other hand, azelaic acid with two terminal carboxylic groups forms only hydrogen bonds with the enzyme-active site, beside some weak van der Waals interactions.

## 4. Conclusions

Plants will always remain an open public compounds library filled with template molecules. However, it is our responsibility to venture into them to discover their hidden powers of natural healing. Herein, the untapped mangrove plant *B. gymnorhiza* showed promise as a source of agents for managing skin-related diseases, hyperglycaemia, and obesity based on their significantly high biological activities. However, in-depth studies are required to complement our current results to be able to project the plant as a leading source of novel drugs. Validating the biological activities of the mangrove plant using in vivo and in silico models are thus recommended. Herein, *B. gymnorhiza* demonstrated potent antioxidant capacities in terms of radical scavenging, reducing potential, and chelating power. Between extraction solvents and different plant parts used, multivariate analysis concluded that the extraction solvents exerted more influence on the studied biological activities. The results of docking simulation were in agreement with the findings of the experimental sections. Among the docked compounds, luteolin and azelaic acid showed the highest affinity against lipase and tyrosinase enzymes, respectively. The data collected herein underlines that future work is needed to provide a comprehensive overview of *B. gymnorhiza*’s full pharmacological potential. It is also envisaged that, in future studies, from a cytogenetic point of view, the extracts from different morphological parts of *B. gymnorhiza* should be studied. Further characterization of bioactive compounds is needed using nuclear magnetic resonance (NMR). Additionally, the kinetic studies of the enzymes could be investigated.

## Figures and Tables

**Table 1:** Chemical composition of fruit extracts.

Compound Name	MeOH	Ethyl Acetate
Quinic acid	+	+
Citric acid	+	-
Brugierol	+	+
Gallocatechin (Casuarin, Gallocatechol)	+	+
Protocatechuic acid (3,4-Dihydroxybenzoic acid)	+	-
Neochlorogenic acid (5-O-Caffeoylquinic acid)	+	+
Procyanidin B isomer 1	+	-
Procyanidin B isomer 2	+	-
3-O-(4-Coumaroyl) quinic acid	+	+
Catechin	+	+
Epigallocatechin (Epigallocatechol)	+	+
Chlorogenic acid (3-O-Caffeoylquinic acid)	+	-
3-O-Feruloylquinic acid	+	-
Ampelopsin (Ampeloptin, Dihydromyricetin)	+	+
Procyanidin B isomer 3	+	-
Vanillin	+	+
Chryptochlorogenic acid (4-O-Caffeoylquinic acid)	+	-
Epicatechin	+	+
4-O-(4-coumaroyl) quinic acid	+	-
3-(Benzoyloxy)-2-hydroxypropylglucuronic acid	+	-
4-Coumaric acid	+	+
Antiarol (3,4,5-Trimethoxyphenol)	-	-
Loliolide or Isololiolide	+	+
4-O-Feruloylquinic acid	+	-
Riboflavin	-	-
Indole-3-lactic acid	+	-
Ferulic acid	+	-
Loliolide or Isololiolide	+	+
4-Hydroxy-3-methoxycinnamaldehyde (Coniferyl aldehyde)	+	+
Sinapic acid (Sinapinic acid)	-	-
Myricetin-3-O-rutinoside	+	+
Cinchonain I isomer 1	+	+
Theaflavin	+	-
Dihydrokaempferol (Aromadendrin, Katuranin)	-	-
Cinchonain I isomer 2	+	+
Methoxy-tetrahydroxy(iso)flavone isomer 1	+	+
Isoquercitrin (Hirsutrin, Quercetin-3-O-glucoside)	+	-
Rutin (Quercetin-3-O-rutinoside)	+	-
Myricetin (Cannabisetin, Myricetol, 3,3′,4′,5,5′,7-hexahydroflavone)	-	-
Azelaic acid	+	+
Methoxy-trihydroxy(iso)flavone	+	+
Kaempferol-3-O-rutinoside (Nicotiflorin)	+	-
Gramrione (5,5′-Dimethoxy-3′,4′,7-trihydroxyflavone)	+	+
Dihydroxy-trimethoxy(iso)flavone	+	+
Quercetin (3,3′,4′,5,7-Penthahyroxyflavone)	-	-
Naringenin (4′,5,7-Trihydroxyflavanone)	+	+
Sebacic acid	+	+
Methoxy-tetrahydroxy(iso)flavone isomer 2	+	+
Bruguierol A	+	+
Juniperic acid (16-hydroxyhexadecanoic acid)		
Lupeol caffeate	+	+
Lupeol coumarate	+	+

+: Present; -: Not present.

**Table 2:** Chemical composition of leaf extracts.

Compound Name	MeOH	Ethyl Acetate
Quinic acid	+	+
Brugierol	+	+
Gallocatechin	-	-
Protocatechuic acid (3,4-Dihydroxybenzoic acid)	+	+
Catechol	-	-
Genistic acid (2,5-Dihydroxybenzoic acid)	-	-
Neochlorogenic acid (5-O-Caffeoylquinic acid)	+	-
Procyanidin B isomer 1	-	-
3-O-(4-Coumaroyl) quinic acid	+	-
Catechin	+	-
Epigallocatechin	-	-
Chlorogenic acid (3-O-Caffeoylquinic acid)	+	-
Dihydroxybenzoic acid isomer	-	-
Caffeic acid	+	+
3-O-Feruloylquinic acid	+	-
Procyanidin B	+	-
Vanillin	-	+
Chryptochlorogenic acid (4-O-Caffeoylquinic acid)	+	-
Epicatechin	+	-
4-O-(4-Coumaroyl) quinic acid	+	-
3-(Benzoyloxy)-2-hydroxypropylglucuronic acid	+	-
4-Coumaric acid	+	+
Antiarol (3,4,5-Trimethoxyphenol)	+	+
Loliolide or Isololiolide	+	+
4-O-Feruloylquinic acid	+	-
Riboflavin	+	-
Cinchonain I isomer 1	+	+
Ferulic acid	+	+
Taxifolin (Didydroquercetin)	-	+
Loliolide or Isololiolide	+	+
Dimethoxy-trihydroxy(iso)flavone-O-hexoside isomer 1	+	-
Dimethoxy-trihydroxy(iso)flavone-O-hexoside isomer	-	-
Isoferulic acid	+	+
Dihydroxy-methoxy(iso)flavone-O-hexoside	+	-
Quercetin-O-dirhamnosylhexoside	+	-
Myricetin-3-O-rutinoside	+	+
Cinchonain I isomer 2	+	+
Kaempferol-O-dirhamnosylhexoside	+	-
Cinchonain I isomer 3	+	+
Methoxy-tetrahydroxy(iso)flavone isomer 1	+	+
Isoquercitrin (Hirsutrin, Quercetin-3-O-glucoside)	+	+
Dimethoxy-trihydroxy(iso)flavone-O-hexoside isomer 2	+	-
Rutin (Quercetin-3-O-rutinoside)	+	+
Apigenin-O-rhamnosylhexoside	+	-
Azelaic acid	+	+
Methoxy-trihydroxy(iso)flavone	+	+
Kaempferol-3-O-rutinoside (Nicotiflorin)	+	+
Cinchonain I isomer 4	+	+
Gramrione (5,5′-Dimethoxy-3′,4′,7-trihydroxyflavone)	+	+
Dimethoxy-trihydroxy(iso)flavone-O-hexoside isomer 3	+	+
Dihydroxy-methoxy(iso)flavone	+	+
Dihydroxy-dimethoxy(iso)flavone	+	+
Dihydroxy-trimethoxy(iso)flavone	+	+
Quercetin (3,3′, 4′, 5, 7-Pentahydroxylflavone)	-	+
Naringenin (4′,5,7-Trihydroxyflavanone)	+	+
Luteolin (3′,4′,5,7-Tetrahydroxyflavone)	+	+
Methoxy-tetrahydroxy(iso)flavone isomer 2	+	+
Dimethoxy-trihydroxy(iso)flavone-O-rhamnoside	+	+
Kaempferol (3,4′,5,7-Tetrahydroxyflavone)	+	+
Apigenin (4′,5,7-Trihydroxyflavone)	+	+
Tricin (3′,5′-Dimethoxy-4′,5,7-trihydroxyflavone)	+	+
Bruguierol A	+	+

+: Present; -: Not present.

**Table 3:** Chemical composition of root extracts.

Compound Name	MeOH	Ethyl Acetate
Quinic acid	+	+
Citric acid	+	-
Brugierol	+	-
Gallocatechin	+	+
Unidentified compound	-	-
Protocatechuic acid (3,4-Dihydroxybenzoic acid)	+	+
Neochlorogenic acid (5-O-Caffeoylquinic acid)	+	-
Syringic acid-O-hexoside isomer 1	+	-
Syringic acid-O-hexoside isomer 1	+	-
Prodelphinidin C	+	-
Unidentified compound	-	-
Catechin	+	+
Epigallocatechin	+	+
Chlorogenic acid (3-O-Caffeoylquinic acid)	+	-
Caffeic acid	+	+
Unidentified compound	-	+
Procyanidin B isomer 1	+	+
Vanillin	+	+
Syringic acid	-	+
Chryptochlorogenic acid (4-O-Caffeoylquinic acid)	+	-
Procyanidin C	+	+
Epicatechin	+	+
4-Coumaric acid	-	+
3-(Benzoyloxy)-2-hydroxypropylglucuronic acid	+	-
Antiarol (3,4,5-Trimethoxyphenol)	+	+
3,4-Dihydro-3-hydroxy-7-methoxy-2H-1,5-benzodithiepine-6,9-dione	+	+
Cinchonain I isomer 1	+	+
Epiafzelechin	+	+
Ferulic acid	+	+
Taxifolin (Dihydroquercetin)	+	+
Isoferulic acid	-	+
4-Hydroxy-3-methoxycinnamaldehyde (Coniferyl aldehyde)	+	+
Procyanidin B isomer 2	+	-
Trihydroxystilbene	+	+
Myricetin-3-O-rutinoside	+	-
Cinchonain I isomer 2	+	+
Cinchonain I isomer 3	+	+
Rutin (Quercetin-3-O-rutinoside)	+	+
Azelaic acid	+	+
Methoxy-trihydroxy(iso)flavone	+	+
Kaempferol-3-O-rutinoside (Nicotiflorin)	-	+
Cinchonain I isomer 4	+	+
Gramrione (5,5′-Dimethoxy-3′,4′,7-trihydroxyflavone)	+	+
Quercetin (3,3′,4′,5,7-Pentahydroxyflavone)	-	+
Naringenin (4′,5,7-Trihydroxyflavanone)	-	+
3-O-Methylellagic acid-4′-O-rhamnoside	+	-
Sebacic acid	+	+
Phloretin	+	+
Tricin (3′,5′-Dimethoxy-4′,5,7-trihydroxyflavone)	+	+
Norstictic acid	+	+
Methyl-trihydroxyxanthone	+	+
16,17-Dihydroxy-9(11)-kauren-19-al or Steviol	+	+
1-Hydroxy-8(14)-isopimaren-1,15,16-triol or isomer	+	+
13-Hydroxy-16-kauren-19-al or isomer	+	+
Bruguierol A	+	+
1-Hydroxy-8(14)-isopimaren-1,15,16-triol or isomer	+	+
Dihydroxy-methoxy-methylxanthone	+	+
1-Hydroxy-8(14)-isopimaren-1,15,16-triol or isomer	+	+
13-Hydroxy-16-kauren-19-al or isomer	+	+
Unidentified xanthone isomer 1	+	+
Unidentified xanthone isomer 2	+	+
Isopimar-7-en-15,16-diol or isomer	+	+
Isopimar-7-en-15,16-diol or isomer	+	+

+: Present; -: Not present.

**Table 4:** Chemical composition of twig extracts.

Compound Name	MeOH	Ethyl Acetate
Quinic acid	+	+
Citric acid	+	+
Brugierol	+	+
Gallocatechin	+	-
Protocatechuic acid (3,4-Dihydroxybenzoic acid)	+	+
Neochlorogenic acid (5-O-Caffeoylquinic acid)	+	-
Syringic acid-O-hexoside isomer 1	+	+
Syringic acid-O-hexoside isomer 2	+	+
Catechin	+	-
Epigallocatechin	+	-
Chlorogenic acid (3-O-Caffeoylquinic acid)	+	-
Caffeic acid	+	+
Vanillin	-	+
Procyanidin B	+	-
Chryptochlorogenic acid (4-O-Caffeoylquinic acid)	+	-
Syringic acid	+	+
Ehyl syringate	-	+
Procyanidin C	+	-
Epicatechin	+	-
3-(Benzoyloxy)-2-hydroxypropylglucuronic acid	+	-
4-Coumaric acid	+	+
Antiarol (3,4,5-Trimethoxyphenol)	+	+
3,4-Dihydro-3-hydroxy-7-methoxy-2H-1,5-benzodithiepine-6,9-dione	+	+
Riboflavin	+	-
Cinchonain I isomer 1	+	-
Ferulic acid	+	+
Loliolide or Isololiolide	+	+
Coumarin	-	+
4-Hydroxy-3-methoxycinnamaldehyde (Coniferyl aldehyde)	-	+
3,5-Dimethoxy-4-hydroxycinnamaldehyde (Sinapyl aldehyde)	-	+
Isoquercitrin (Hirsutin, Quercetin-3-O-glucoside)	-	+
Cinchonain I isomer 2	+	-
Cinchonain I isomer 3	+	-
Rutin (Quercetin-3-O-rutinoside)	+	-
Azelaic acid	+	+
Methoxy-trihydroxy(iso)flavone	+	+
Kaempferol-3-O-rutinoside (Nicotiflorin)	+	-
Cinchonain I isomer 4	+	-
Gramrione (5,5′-Dimethoxy-3′,4′,7-trihydroxyflavone)	+	+
Dihydroxy-methoxy(iso)flavone	+	+
Dihydroxy-dimethoxy(iso)flavone	+	+
Dihydroxy-trimethoxy(iso)flavone	+	+
Naringenin (4′,5,7-Trihydroxyflavanone)	+	+
16,17-Dihydroxy-9(11)-kauran-19-al	+	+
16,17-Dihydroxy-9(11)-kauren-19-al or Steviol	+	+
1-Hydroxy-8(14)-isopimaren-1,15,16-triol or isomer	+	+
Methyl 16α,17-dihydroxy-9(11)-kauren-19-oate or isomer	+	+
13-Hydroxy-16-kauren-19-al or isomer	+	+
16,17-Dihydroxy-9(11)-kauran-19-al isomer	+	+
Bruguierol A	+	+
Methyl 16α,17-dihydroxy-9(11)-kauren-19-oate or isomer	+	+
1-Hydroxy-8(14)-isopimaren-1,15,16-triol or isomer	+	+
1-Hydroxy-8(14)-isopimaren-1,15,16-triol or isomer	+	+
13-Hydroxy-16-kauren-19-al or isomer	+	+
Isopimar-7-en-15,16-diol or isomer	+	+
Isopimar-7-en-15,16-diol or isomer	+	+
Lupeol caffeate	+	-
Lupeol coumarate	+	-

+: Present; -: Not present.

**Table 5:** Extraction yield (%) and total bioactive compounds of *Bruguiera gymnorhiza* extracts.

	Yield (%)	Total Phenolic Acid (mg CAE/g)	Total Phenolic Content (mg GAE/g)	Total Flavonoid Content (mg RE/g)	Total Flavanol (mg CE/g)	Total Tannin (mg CE/g)	Total Triterpenoid (mg OAE/g)
**BLM**	12.62	1.21 ± 0.08 ^d,e^	30.26 ± 0.25 ^f^	9.28 ± 0.09 ^c^	9.72 ± 0.17 ^e^	17.95 ± 0.35 ^e^	9.12 ± 0.30 ^c^
**BRM**	9.60	9.86 ± 0.37 ^a^	161.64 ± 1.45 ^b^	1.92 ± 0.07 ^f^	119.00 ± 1.49 ^b^	134.55 ± 5.97 ^b^	55.73 ± 6.48 ^a^
**BTM**	8.26	5.22 ± 0.23 ^b^	91.06 ± 0.98 ^c^	1.65 ± 0.16 ^f^	34.84 ± 0.68 ^d^	48.21 ± 1.71 ^d^	21.19 ± 1.63 ^b^
**BFM**	14.28	0.77 ± 0.11 ^e^	174.18 ± 4.27 ^a^	3.29 ± 0.05 ^e^	71.99 ± 2.46 ^d^	176.24 ± 3.10 ^a^	63.11 ± 3.27 ^a^
**BLE**	1.88	1.95 ± 0.73 ^d^	54.94 ± 0.88 ^e^	20.39 ± 0.43 ^b^	5.67 ± 0.01 ^f^	8.66 ± 1.25 ^f^	13.30 ± 0.12 ^c^
**BRE**	0.46	3.33 ± 0.38 ^c^	71.08 ± 0.39 ^d^	1.27 ± 0.07 ^f^	56.81 ± 1.26 ^c^	92.37 ± 2.10 ^c^	24.37 ± 0.42 ^b^
**BTE**	1.06	0.85 ± 0.05 ^e^	27.27 ± 0.43 ^f^	4.48 ± 0.08 ^d^	1.53 ± 0.04 ^g^	5.99 ± 0.55 ^f^	6.84 ± 0.30 ^c^
**BFE**	0.66	0.73 ± 0.15 ^e^	52.52 ± 0.37 ^e^	36.64 ± 0.59 ^a^	8.61 ± 0.09 ^e,f^	7.56 ± 0.60 ^f^	9.76 ± 0.11 ^c^

Different letters (^a–g^) indicate statistically significant differences in the tested extracts (*p* < 0.05). Values are expressed as mean ± S.D. of three parallel measurements. Abbreviations: BLM: *Bruguiera* leaf methanolic; BRM: *Bruguiera* root methanolic; BTM: *Bruguiera* twig methanolic; BFM: *Bruguiera* fruit methanolic; BLE: *Bruguiera* leaf ethyl acetate; BRE: *Bruguiera* root ethyl acetate; BTE: *Bruguiera* twig ethyl acetate; BFE: *Bruguiera* fruit ethyl acetate; GAE: Gallic acid equivalents; RE: Rutin equivalent; CE: Caffeic acid equivalent; OAE: Oleanolic acid equivalent.

**Table 6:** Antioxidant properties of *B. gymnorhiza* extracts.

	DPPH (mg TE/g)	ABTS (mg TE/g)	CUPRAC (mg TE/g)	FRAP (mg TE/g)	Phosphomolybdenum (mmol TE/g)	Chelating Activity (mg EDTAE/g)
**BLM**	42.35 ± 0.36 ^d^	63.73 ± 0.58 ^e^	108.56 ± 1.63 ^g^	65.51 ± 0.81^d^	0.69 ± 0.06 ^e^	33.84 ± 0.72 ^b^
**BRM**	439.53 ± 1.52 ^b^	470.58 ± 7.68 ^a^	858.28 ± 20.92 ^b^	513.48 ± 19.84 ^b^	3.65 ± 0.02 ^b^	8.20 ± 0.92 ^e^
**BTM**	97.04 ± 0.35 ^c^	140.56 ± 0.20 ^c^	321.27 ± 2.63 ^c^	185.72 ± 3.03 ^c^	1.86 ± 0.13 ^d^	11.61 ± 0.79 ^d^
**BFM**	492.62 ± 5.31 ^a^	457.88 ± 8.32 ^b^	961.46 ± 11.18 ^a^	552.49 ± 8.71 ^a^	4.17 ± 0.31 ^a^	11.92 ± 0.38 ^d^
**BLE**	19.85 ± 0.57 ^e^	26.22 ± 1.02 ^f^	144.92 ± 2.05 ^f^	59.22 ± 0.91 ^d^	2.68 ± 0.15 ^c^	6.07 ± 0.92 ^f^
**BRE**	94.07 ± 0.25 ^c^	140.62 ± 0.10 ^c^	243.82 ± 1.75 ^d^	165.01 ± 3.64 ^c^	2.26 ± 0.13 ^c,d^	4.87 ± 0.62 ^f^
**BTE**	12.73 ± 0.26 ^f^	13.77 ± 1.25 ^g^	72.60 ± 1.28 ^h^	34.23 ± 1.01 ^e^	1.10 ± 0.07 ^e^	16.87 ± 0.76 ^c^
**BFE**	42.54 ± 0.50 ^d^	98.17 ± 2.08 ^d^	187.18 ± 4.07 ^e^	53.16 ± 0.44 ^d,e^	2.54 ± 0.23 ^c^	60.22 ± 0.05 ^a^

Different letters (^a–h^) indicate statistically significant differences in the tested extracts (*p* < 0.05). Values are expressed as mean ± S.D. of three parallel measurements. Abbreviations: BLM: *Bruguiera* leaf methanolic; BRM: *Bruguiera* root methanolic; BTM: *Bruguiera* twig methanolic; BFM: *Bruguiera* fruit methanolic; BLE: *Bruguiera* leaf ethyl acetate; BRE: *Bruguiera* root ethyl acetate; BTE: *Bruguiera* twig ethyl acetate; BFE: *Bruguiera* fruit ethyl acetate; TE: Trolox equivalent; EDTAE: EDTA equivalent.

**Table 7:** Enzyme inhibitory effects of *B. gymnorhiza* extracts.

	AChE Inhibition (mg GALAE/g)	BChE Inhibition (mg GALAE/g)	Tyrosinase Inhibition (mg KAE/g)	Amylase Inhibition (mmol ACAE/g)	Glucosidase Inhibition (mmol ACAE/g)	Elastase Inhibition (mg CAE/g)	Lipase Inhibition (mg OE/g)
**BLM**	4.23 ± 0.15 ^c^	1.60 ± 0.09 ^d^	133.63 ± 0.43 ^d^	0.42 ± 0.02 ^d^	na	3.63 ± 0.03 ^c,d^	79.37 ± 2.29 ^b,c^
**BRM**	4.95 ± 0.02 ^a,b^	4.60 ± 0.04 ^b,c^	147.72 ± 0.86 ^b^	0.76 ± 0.01 ^b^	na	4.61 ± 0.06 ^a^	88.81 ± 4.54 ^b^
**BTM**	4.80 ± 0.12 ^a,b^	3.54 ± 0.04 ^c^	139.77 ± 0.26 ^c^	0.61 ± 0.01 ^c^	na	4.24 ± 0.08 ^a,b^	76.37 ± 4.29 ^b–d^
**BFM**	4.68 ± 0.08 ^b,c^	5.17 ± 0.11 ^b^	155.35 ± 0.29 ^a^	1.00 ± 0.05 ^a^	na	4.56 ± 0.10 ^a^	69.49 ± 8.22 ^c,d^
**BLE**	3.40 ± 0.41 ^d^	3.52 ± 1.05 ^c^	125.21 ± 1.59 ^e^	0.96 ± 0.02 ^a^	22.90 ± 0.81 ^c^	3.93 ± 0.25 ^b,c^	102.80 ± 7.25 ^a^
**BRE**	4.64 ± 0.24 ^b,c^	5.64 ± 0.72 ^a,b^	134.73 ± 3.29 ^d^	0.70 ± 0.03 ^b^	30.43 ± 0.07 ^a^	3.44 ± 0.29 ^d^	64.30 ± 1.92 ^d^
**BTE**	5.34 ± 0.17 ^a^	6.90 ± 0.10 ^a^	123.65 ± 0.39 ^e^	0.73 ± 0.04 ^b^	30.98 ± 0.01 ^a^	3.91 ± 0.19 ^b,c^	75.34 ± 1.35 ^c,d^
**BFE**	na	0.87 ± 0.03 ^d^	33.48 ± 0.43 ^f^	0.93 ± 0.04 ^a^	28.59 ± 0.08 ^b^	4.08 ± 0.09 ^b,c^	na

Different letters (^a–f^) indicate statistically significant differences in the tested extracts (*p* < 0.05). Values are expressed as mean ± S.D. of three parallel measurements. Abbreviations: BLM: *Bruguiera* leaf methanolic; BRM: *Bruguiera* root methanolic; BTM: *Bruguiera* twig methanolic; BFM: *Bruguiera* fruit methanolic; BLE: *Bruguiera* leaf ethyl acetate; BRE: *Bruguiera* root ethyl acetate; BTE: *Bruguiera* twig ethyl acetate; BFE: *Bruguiera* fruit ethyl acetate; GALAE: Galantamine equivalent; KAE: Kojic acid equivalent; ACAE: Acarbose equivalent; OE: Orlistat equivalent; CAE: Catechin equivalent; na: not active.

**Table 8:** Selected docked bioactive compounds.

Enzyme	Compound	Chemical Structure
**Lipase**	Luteolin	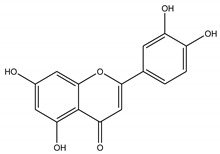
	Taxifolin	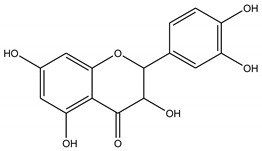
	Bruguierol A	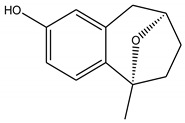
	Brugierol	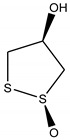
**Tyrosinase**	Azelaic acid	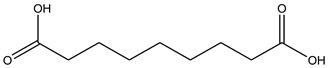
	Quinic acid	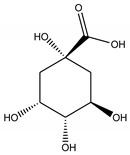

**Table 9:** Docking free energy and inhibition constants for the docked compounds.

Enzyme	Compounds	Binding Free Energy (kcal/mol)	Inhibition Constant (K_i_)
**Lipase**	Luteolin	-8.49	600.10 nM
	Taxifolin	-7.02	7.11 µM
	Bruguiera A	-7.14	5.83 µM
	Brugierol	-3.14	4.96 mM
**Tyrosinase**	Azelaic acid	-5.35	120.45 µM
	Quinic acid	-4.57	443.43 µM

## Abbreviations

BLM: *Bruguiera* leaf methanolic; BRM: *Bruguiera* root methanolic; BTM: *Bruguiera* twig methanolic; BFM: *Bruguiera* fruit methanolic; BLE: *Bruguiera* leaf ethyl acetate; BRE: *Bruguiera* root ethyl acetate; BTE: *Bruguiera* twig ethyl acetate; BFE: *Bruguiera* fruit ethyl acetate.
